# Supplementary material for: Gonadotrophs have a dual origin, with most derived from early postnatal pituitary stem cells
Source: Nat Commun. 2025 May 21;16:4280. doi: 10.1038/s41467-025-59495-7 (PMC12095566; doi:10.1038/s41467-025-59495-7)
Supplement: Supplementary file 1 — Supplementary Information [file 41467_2025_59495_MOESM1_ESM.pdf]

Supplementary Figures

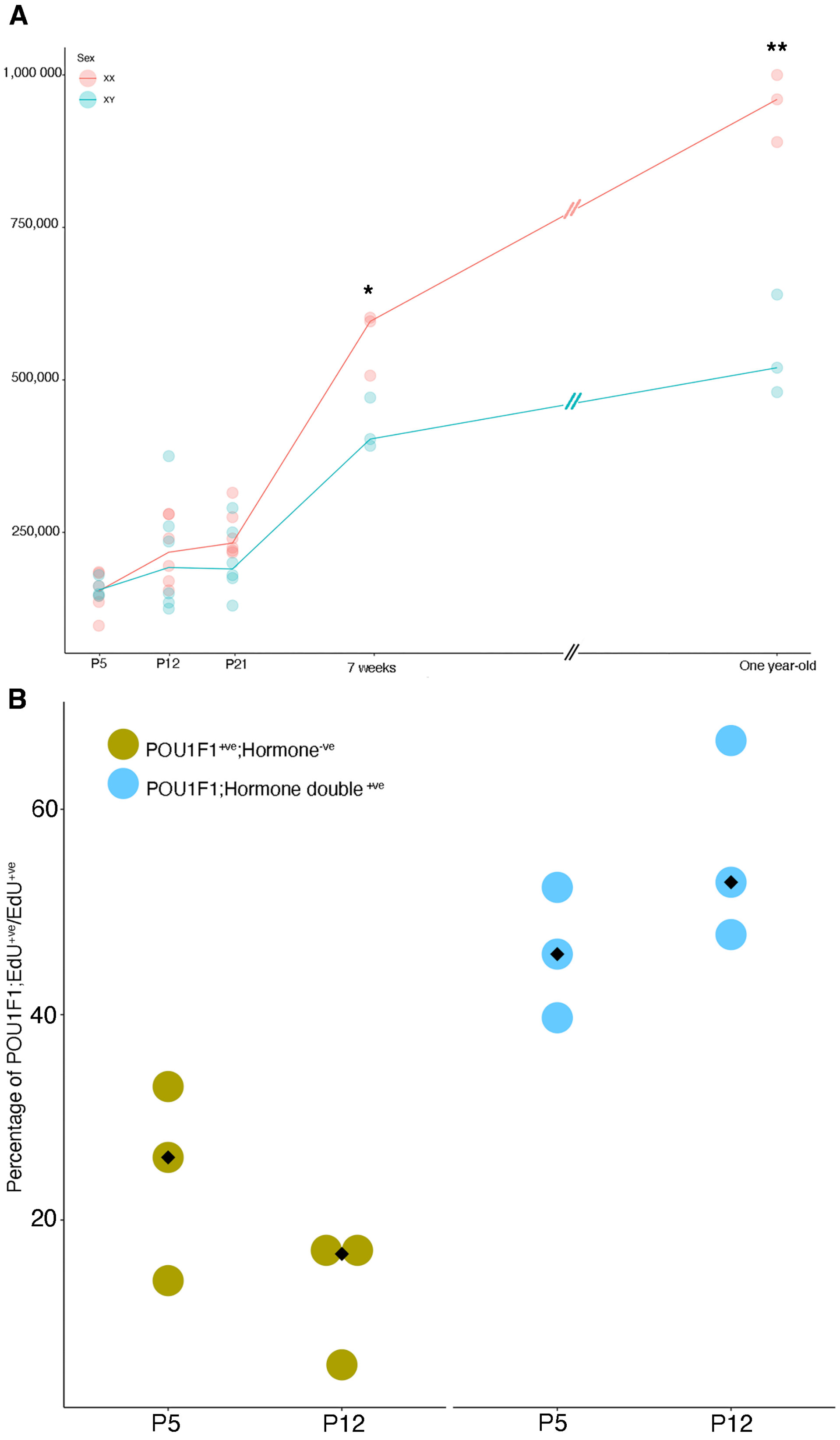

Supplementary Fig.1: Quantification of total pituitary cell numbers and POU1F1^{+ve} cell proliferation.

A) Total anterior lobe pituitary cell numbers were counted after dissociation (Supplementary Table 2); the line indicates the median value. At 7 weeks and up to one-year, female pituitaries contain significantly more cells (unpaired t-tests, $p = 0.0206$ and $p = 0.0022$ respectively). B) Proliferation was assessed by quantifying the number of EdU^{+ve} cells in male mice pituitaries after a one-hour pulse at the ages indicated. Quantification was performed manually on sections stained by immunofluorescence for POU1F1 and a mix of rabbit antibodies recognising all POU1F1 lineage endocrine cell types (GH, Prl, TSH). A comparable percentage of hormone^{+ve} and hormone^{-ve} POU1F1^{+ve} cells proliferate at P5 and P12 with no significant difference between timepoints or cell type. Each dot represents one animal, the diamond in B represents the median value. Source data are provided as a Source Data file.

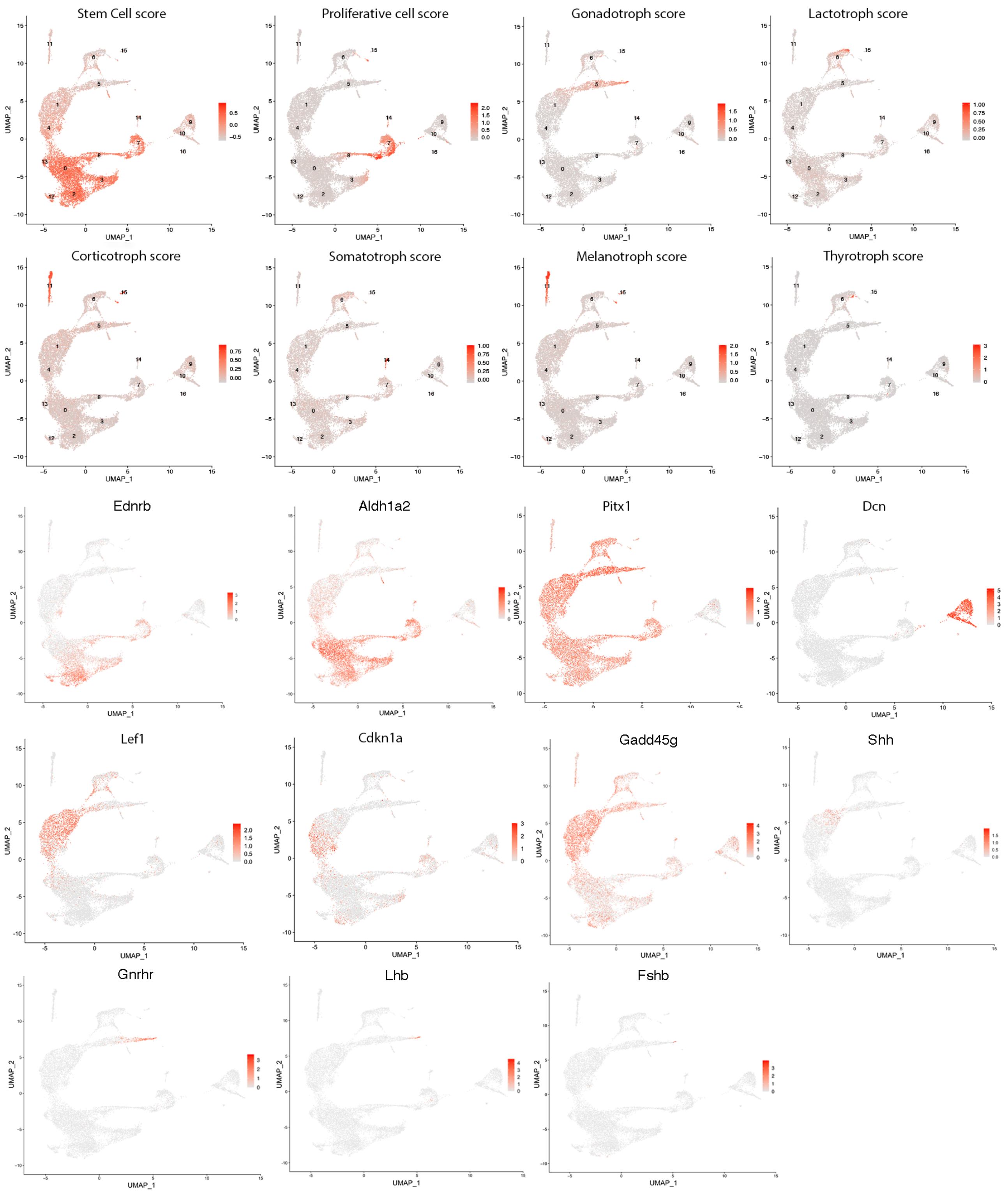

<u>Supplementary Fig.2</u>: UMAP representation of cell type scores and selected marker expression. We used the same cell type signatures (Supplementary Table 6) as previously (*22*).

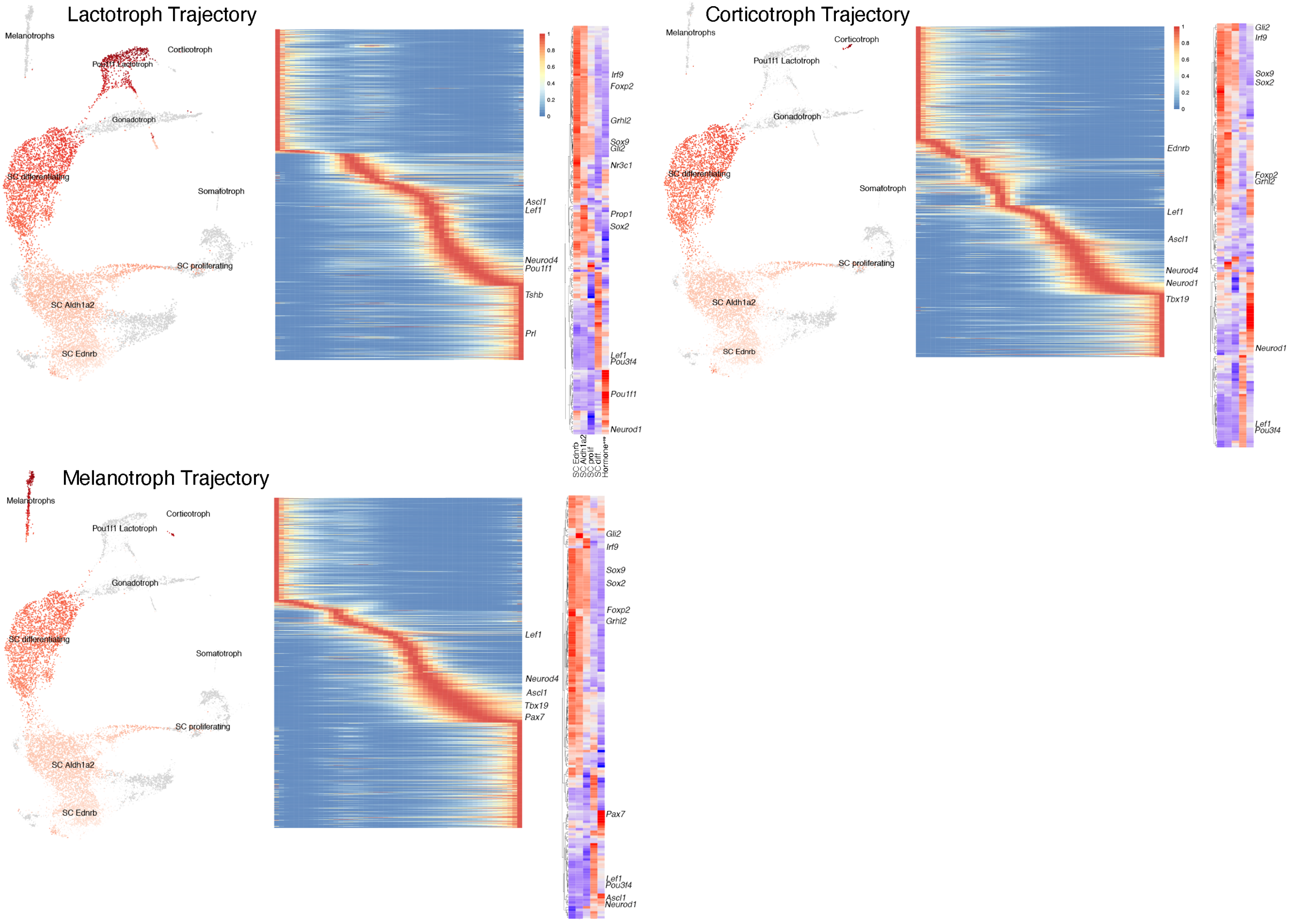

<u>Supplementary Fig.3</u>: Slingshot trajectories and heatmaps for lactotroph, corticotroph and melanotroph trajectories.

Genes common to all trajectories are highlighted. The trajectories presented include endocrine lineages to which SCs contribute, as demonstrated by lineage tracing analyses (Fig.3).

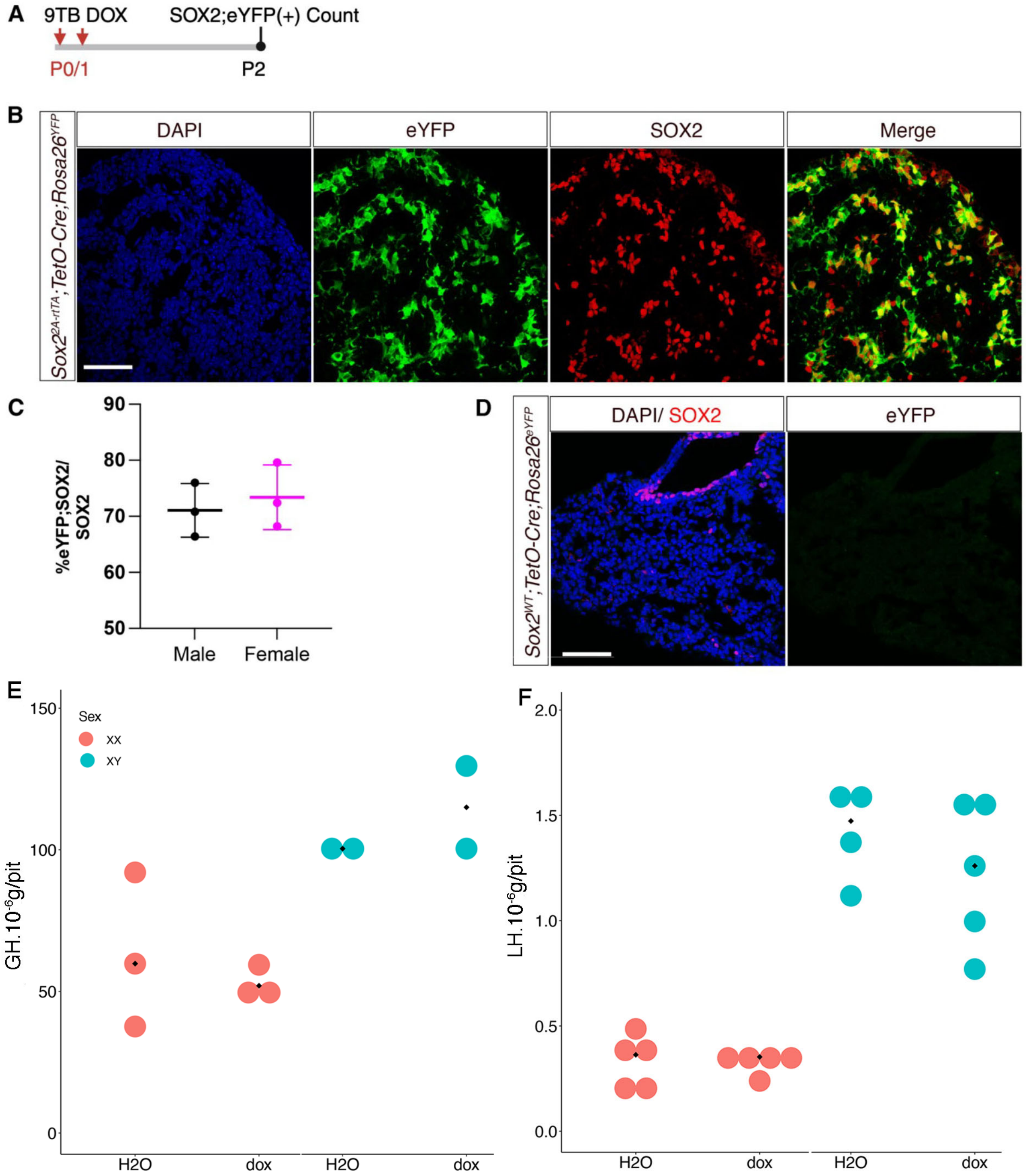

Supplementary Fig.4: Characterisation of the Sox2rtTA allele.

A) Timeline for *Sox2rtTA;eYFP* efficiency assessment. 9TB-Dox was administered at P0 and P1 and the percentage of SOX2;eYFP double^{+ve} cells counted at P2. **B**) Immunostaining for eYFP and SOX2 shows that most SOX2^{+ve} cells express eYFP. The experiment was performed at least three times on independent samples. **C**) Percentage of eYFP;SOX2 double^{+ve} cells as a proportion of the total number of SOX2^{+ve} cells; recombination efficiency is approximately 70% (N=3 per sex). **D**) No expression of eYFP is detected in the absence of the *Sox2rtTA* allele.

E,F) 9TB-Dox or vehicle (water) was injected on two consecutive days between P0 and P4. Pituitaries were harvested at 7 weeks and GH (E) and LH (F) levels measured by RIA. There was no significant difference in LH and GH levels between treated and control animals (Mann-Whitney test, GH p= 0.67 for males and 0.70 for females LH p= 0.41 for males and 0.84 for females). Each dot represents one animal, and the median value is represented. Scale bars represent 50 μm. Source data are provided as a Source Data file.

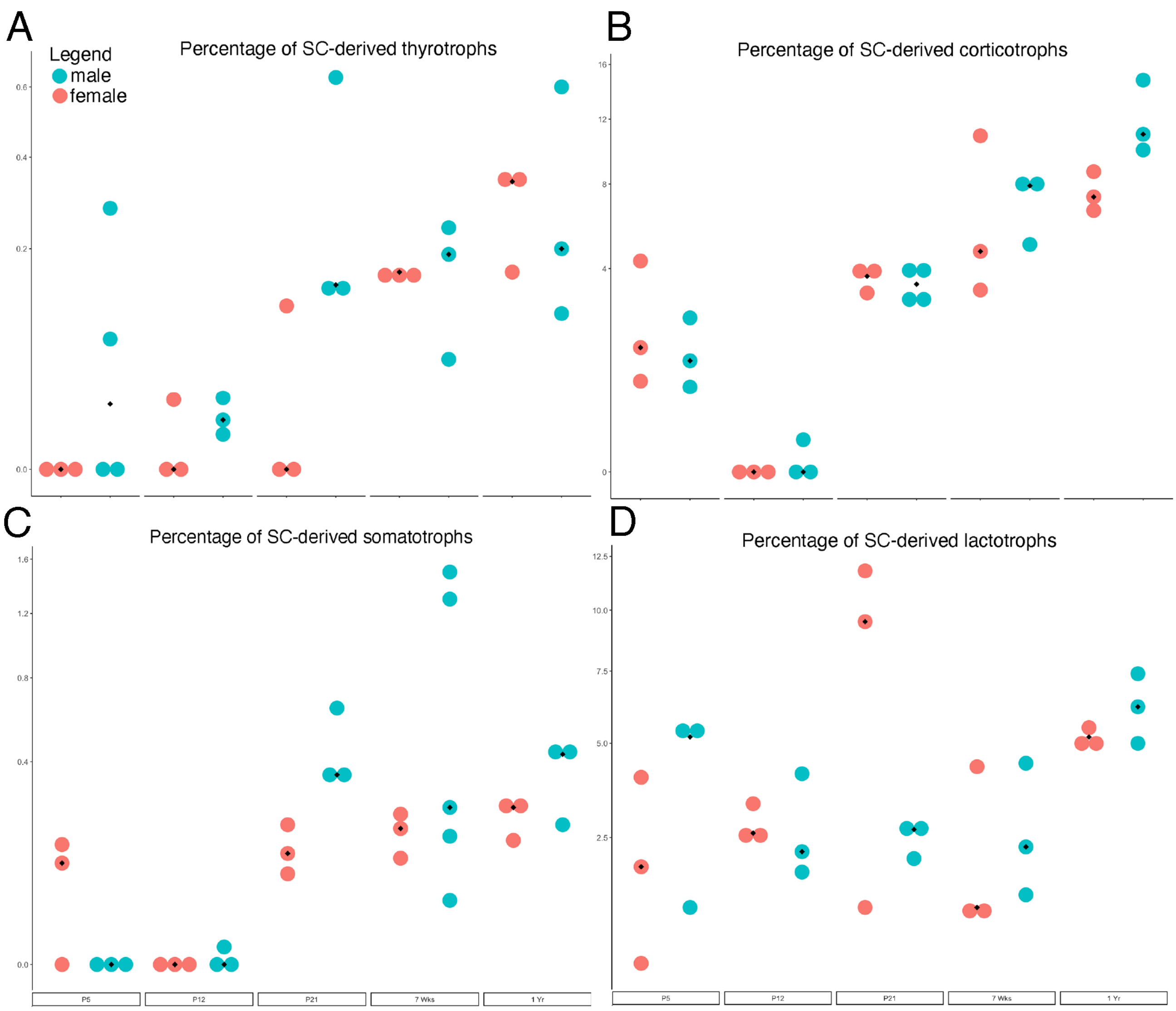

<u>Supplementary Fig.5:</u> Timeline of SC-derived endocrine cell emergence.
As described in Fig.3 for gonadotrophs, pituitaries from lineage traced *Sox2rtTA;eYFP* animals induced at birth were dissociated at the age indicated, cells plated and immunofluorescence for eYFP and each anterior pituitary hormone performed. Automated cell counts were executed as described in Fig.1 and the percentage of SC-derived progeny represented for each endocrine population (eYFP;hormone double $^{+ve}$/total hormone). Beside gonadotrophs (Fig.3) SCs mostly contribute to the POMC lineage (corticotrophs and probably some melanotrophs carried over when the IL was dissected out, **B**), and lactotrophs (PRL, **D**). There is very little contribution to somatotrophs (GH, **C**) and thyrotrophs (TSH, **A**). Each dot represents one animal, and the median value is represented. Source data are provided as a Source Data file.

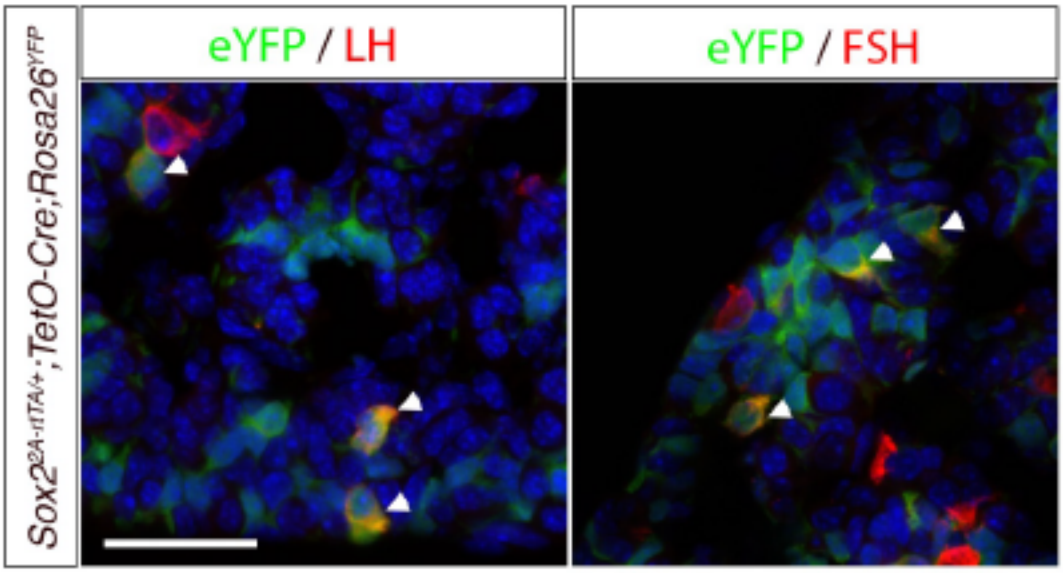

<u>Supplementary Fig.6:</u> Newly differentiated gonadotrophs express both LH and FSH. Double immunofluorescences for eYFP and LH and eYFP and FSH in a P4 female pituitary. 9TBD induction was performed at P0 and P1. Some cells in the progeny of SOX2^{+ve} SC have already acquired a gonadotroph fate with both LH and FSH being upregulated. The experiment was performed at least three times on independent samples. The scale bar represents 30μm.

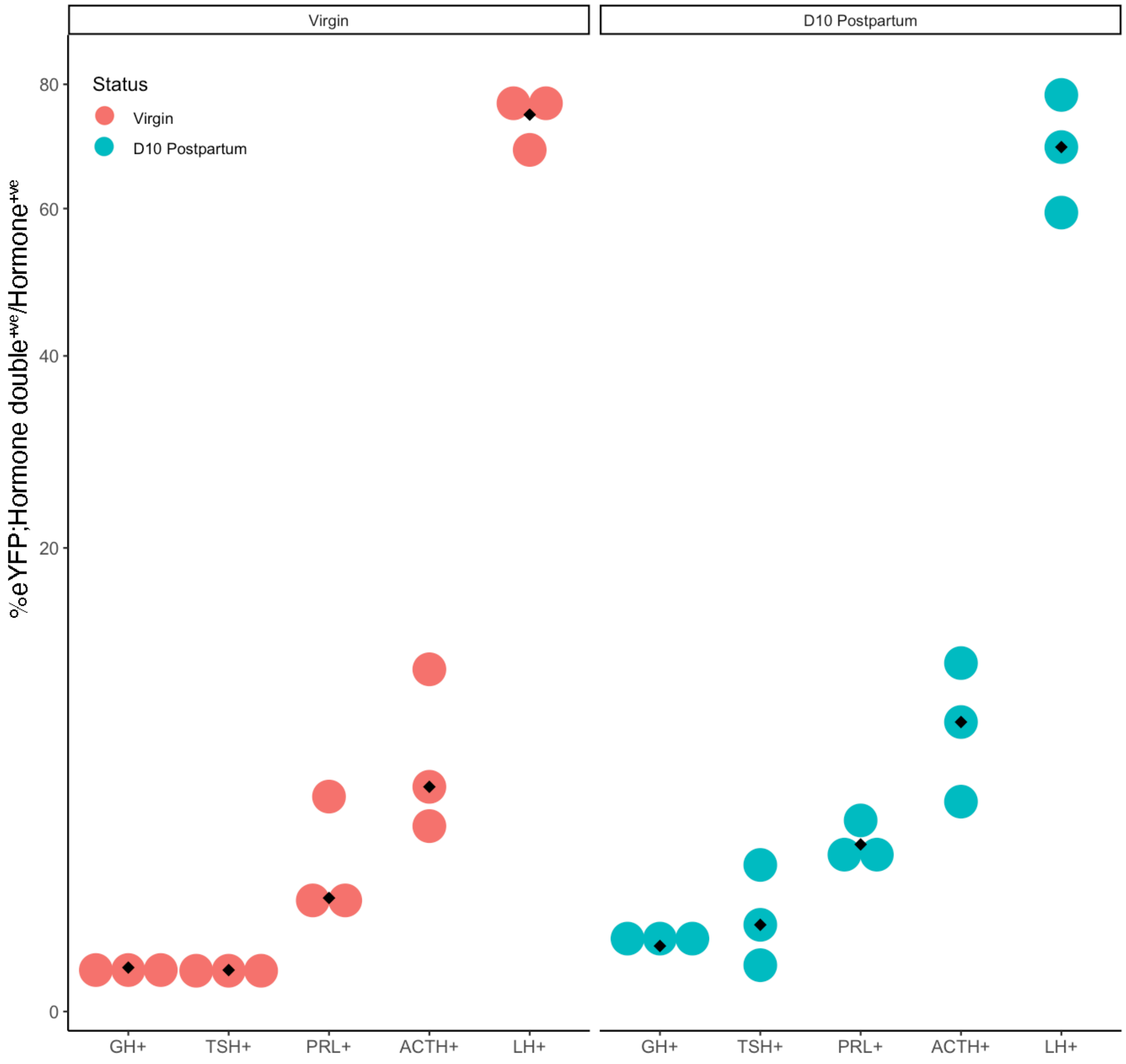

Supplementary Fig.7: Effect of pregnancy and lactation on female pituitary SC endocrine cell contribution on day 10 postpartum.

Lineage tracing of *Sox2rtTA;eYFP* female pups was initiated at P0/P1 and animals mated at 7 weeks. Following their first litter at approximately 10 weeks, pituitaries were harvested from lactating dams on day 10 postpartum and the percentage of eYFP;hormone double^{+ve} cells counted for each cell type. Multiple two-tailed unpaired t-tests were performed between virgin and lactating mice, and the Holm-Šídák method used to correct for multiple comparisons. No significant difference in SC contribution was observed between virgin and lactating females. Each dot represents one animal, and the median value is indicated. Source data are provided as a Source Data file.

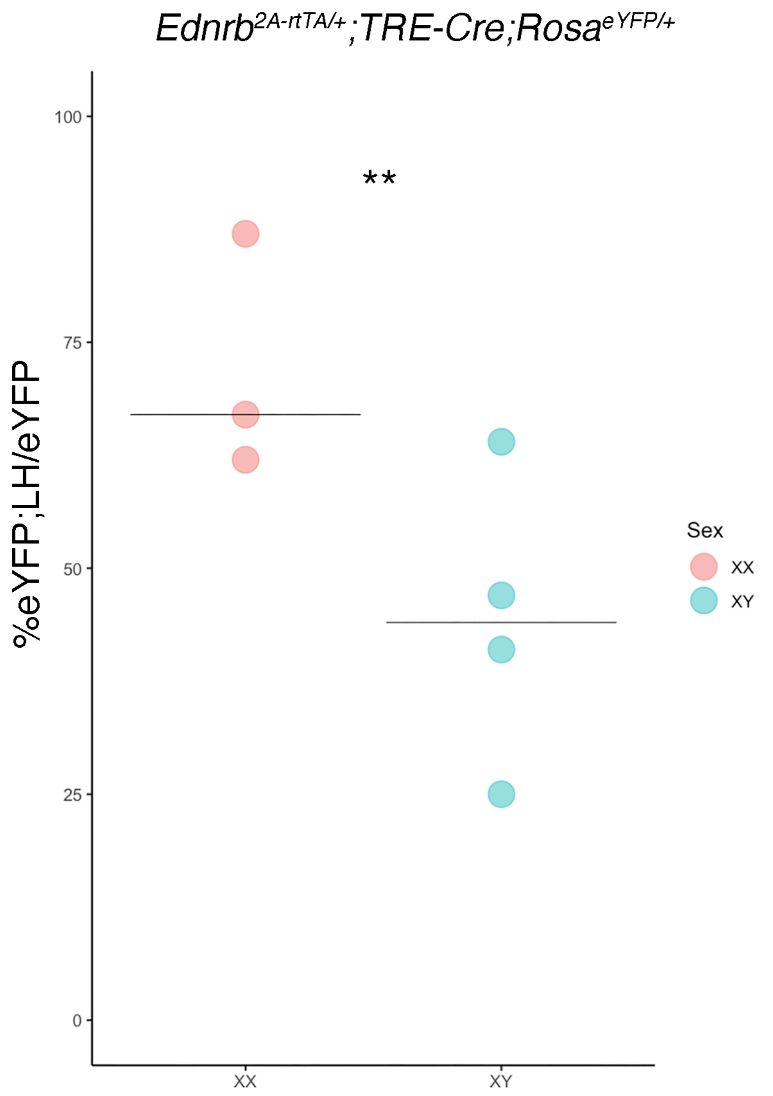

<u>Supplementary Fig.8</u>: Cleft SC lineage tracing in *Ednrb$^{2A\text{-}rtTA/+}$;TetO-Cre;Rosa26$^{ReYFP/+}$* animals.

Pups were treated at P0 and P1 using 9TB-Dox, in the same conditions as *Sox2rtA;eYFP* animals and pituitaries harvested at or after 7 weeks. Tracing was relatively inefficient, probably reflecting the low levels of EDNRB observed *in vivo* (*22*). Traced endothelial cells, morphologically recognisable, were not included in the analysis. In agreement with the data obtained in Sox2rtTA;eYFP analyses, gonadotrophs were mostly represented in EDNRB progeny. Beta regression was used to assess the effect of sex on proportion of LH positive cells in the endocrine progeny of EDNRB^{+ve} cells ($p = 0.007629385$, beta regression) and showed that there are more LH^{+ve} cells in the progeny of female cleft SCs. However, the number of eYFP;LH^{+ve} cells detected per animal was low (between 8 and 37) therefore more data are needed to validate this sex difference. Each dot represents one animal, and the median value is indicated. Source data are provided as a Source Data file.

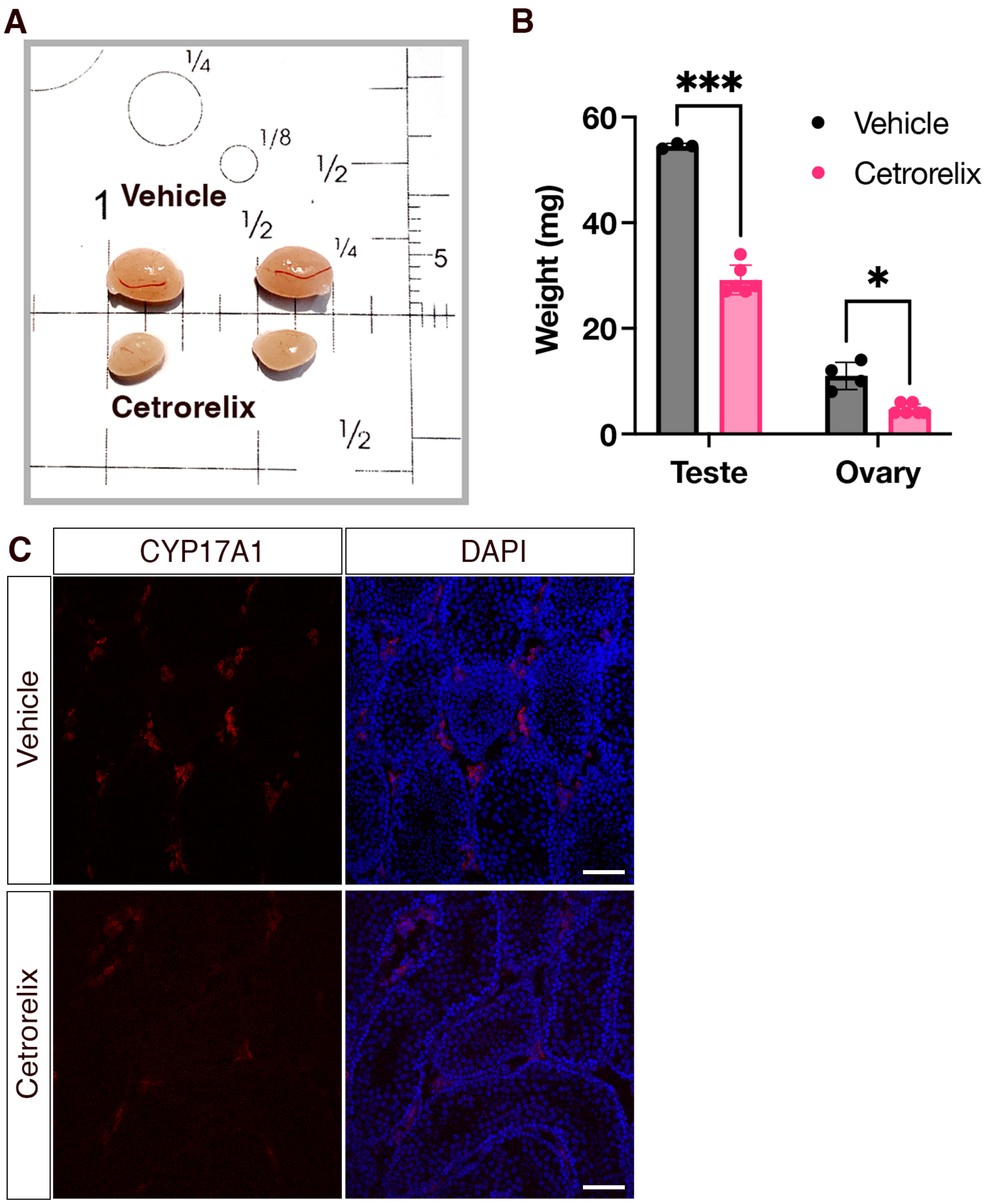

<u>Supplementary Fig.9</u>: Cetrorelix-induced gonadal hypogonadism.
A) Following cetrorelix treatment, we observe at P33 a reduction of testes size compared to vehicle treated controls. **B**) This difference was quantified by weighing both testes and ovaries of treated animals; these showed a reduction in weight compared to vehicle-treated controls (Multiple two-tailed unpaired-tests with Holm-Šídák post-hoc test. *P<0.05, ***P<0.001, minimum of N=3 per treatment.). **C**) CYP17A1 immunofluorescence on testis section shows a reduction in cetrorelix treated animals, demonstrating a decrease in Leydig cells steroidogenesis, induced by lack of gonadotrophins. The experiment was performed at least three times on independent samples. Scale bar = 80 µm.

<u>Supplementary Table 1: Percentage of endocrine cells in the maturing postnatal pituitary.</u>

Mean endocrine percentage and standard deviation (STD) reported for each time point, calculated as Hormone;DAPI double $^{+ve}$/DAPI, following removal of the posterior and intermediate lobes (graph shown Fig1A-B). Minimum of N=3 per timepoint. To establish sexually dimorphic proportions, multiple unpaired t-tests (Mann-Whitney) were performed between sexes for each cell type at each time point, and the two-stage step-up (Benjamini, Krieger, and Yekutieli) method used to correct for multiple comparisons with adjusted p value (q value) reported. Significant values bolded (P<0.05). Fold change (FC) and STD of each cell type in relation to the respectives cell type average percentage at P5 is reported (FC = %AgeX/%P5).

Age	Sex	Somatotroph					Lactotroph					Corticotroph				
		%	STD	q Val	FC P5	STD	%	STD	q Val	FC	STD	%	STD	q Val	FC P5	STD
P5	XX	13.34	2.77	0.272	1.00	0.21	0.91	0.62	0.424	1.00	0.68	8.01	2.92	0.03	1.00	0.36
	XY	10.70	1.06		1.00	0.10	0.59	0.37		1.00	0.63	11.65	1.54		1.00	0.13
P12	XX	8.47	1.41	0.398	0.63	0.11	11.70	2.26	0.639	12.86	2.48	9.92	2.05	0.93	1.24	0.26
	XY	13.18	6.18		1.23	0.58	10.77	2.14		18.25	3.62	9.80	2.17		0.84	0.19
P21	XX	37.16	4.28	0.425	2.79	0.32	13.83	2.73	0.451	15.20	3.00	11.94	1.69	**0.02**	1.49	0.21
	XY	35.36	4.00		3.30	0.37	12.58	3.24		21.32	5.49	8.40	2.44		0.72	0.21
7 wks	XX	23.75	3.03	**0.031**	1.78	0.23	34.80	5.35	**0.025**	38.24	5.88	7.15	0.92	0.25	0.89	0.11
	XY	37.40	6.84		3.50	0.64	25.06	1.95		42.47	3.30	8.39	1.43		0.72	0.12
1 year	XX	23.25	8.25	0.328	1.74	0.62	43.21	6.06	**0.025**	47.49	6.66	6.07	0.65	0.93	0.76	0.08
	XY	28.59	5.04		2.67	0.47	25.93	9.45		43.95	16.02	6.26	1.91		0.54	0.16

Age	Sex	Gonadotroph					Thyrotroph				
P5	XX	2.00	0.26	0.543	1.00	0.13	4.91	0.50	0.224	1.00	0.10
	XY	1.57	0.54		1.00	0.34	5.42	0.66		1.00	0.12
P12	XX	1.97	0.70	>0.999	0.98	0.35	3.35	0.73	0.224	0.68	0.15
	XY	1.79	0.21		1.14	0.13	4.06	0.52		0.75	0.10
P21	XX	5.90	0.61	>0.999	2.95	0.31	1.50	0.43	**0.041**	0.31	0.09
	XY	6.05	1.66		3.86	1.06	2.44	0.55		0.45	0.10
7 wks	XX	7.42	3.24	>0.999	3.71	1.62	1.59	0.19	0.224	0.32	0.04
	XY	7.36	3.28		4.69	2.09	2.08	0.63		0.38	0.12
1 year	XX	5.02	1.51	0.083	2.51	0.76	1.16	0.42	0.550	0.24	0.08
	XY	2.92	0.91		1.86	0.58	1.02	0.22		0.19	0.04

Supplementary Table 2: Total number of cells in the maturing postnatal anterior lobe

Mean live cell count and standard deviation (STD) reported for each time point, following removal of the posterior and intermediate lobes (graph shown Fig1A-B). Minimum of N=3 per timepoint.

Age	Sex	Cell Count ($x10^5$)	
		Mean	STD
P5	XX	1.52	0.33
	XY	1.59	0.15
P12	XX	2.20	0.55
	XY	2.13	0.97
P21	XX	2.49	0.39
	XY	2.04	0.57
7 wks	XX	5.68	0.53
	XY	4.22	0.43
1 yr	XX	9.50	0.56
	XY	5.47	0.83

Supplementary Table 3: Percentage of proliferating endocrine cells in the postnatal pituitary.

Mean percentage and standard deviation reported for each time point, calculated as the percentage of Hormone;EdUdouble^{+ve}/Hormone^{+ve}, following removal of the posterior and intermediate lobes (Graph shown Fig.1C). To establish sex differences in proliferation rates across time points, multiple two-tailed unpaired t-tests were performed and the Holm-Šídák method was used to correct for multiple comparisons.

Age	Sex	GH			PRL			POMC		
		%	STD	Adj P Val	%	STD	Adj P Val	%	STD	Adj P Val
**P5**	XX	5.87	1.76	0.792302	3.89	1.47	0.514020	4.50	1.40	0.758315
	XY	4.77	2.57		6.35	2.35		3.83	0.21	
**P12**	XX	3.43	0.12	0.419445	5.77	1.07	0.576953	2.63	0.58	0.749082
	XY	3.07	0.31		5.04	0.35		3.05	0.36	
**P21**	XX	1.00	0.44	0.827545	3.00	0.89	0.576953	0.27	0.15	0.889507
	XY	0.88	0.16		3.63	0.51		0.29	0.06	

Age	Sex	LH			TSH			SOX2		
		%	STD	Adj P Val	%	STD	Adj P Val	%	STD	Adj P Val
**P5**	XX	1.03	0.21	0.11	3.07	0.21	0.10	8.20	0.53	0.04
	XY	0.53	0.25		2.03	0.47		17.23	2.80	
**P12**	XX	0.44	0.13	0.14	0.41	0.14	0.17	8.87	0.95	0.002
	XY	0.63	0.15		0.70	0.20		2.63	0.55	
**P21**	XX	0.00	0.00	N/A	0.90	0.87	0.17	0.80	0.15	0.51
	XY	0.00	0.00		0.11	0.12		0.73	1.27	

Supplementary Table 4: Percentage of endocrine cells at 7 weeks of age generated postnatally from SOX2^{+ve} SCs.

Percentage of eYFP;Hormone double^{+ve}/Hormone in *Sox2rtTA;eYFP***.** No significant difference was observed between sexes in mean SC-contribution for any lineage. Multiple two-tailed unpaired t-tests were performed and the Holm-Šídák method was used to correct for multiple comparisons.

Cell Type	XX		XY		
	**Mean (%)**	**STD**	**Mean (%)**	**STD**	**Adj P val**
**GH**	0.17	0.06	0.65	0.69	0.966
**TSH**	0.16	0.01	0.16	0.10	0.966
**PRL**	2.20	1.82	2.70	1.54	0.922
**POMC**	6.27	4.08	7.00	1.73	0.966
**LH**	74.93	5.03	71.61	10.54	0.966

Supplementary Table 5: Temporal lineage tracing of SC contribution to all lineages. Percentage of eYFP;Hormone double^{+ve}/Hormone in *Sox2rtTA;eYFP*. No significant difference was observed in mean %eYFP-positivity between sexes. Multiple two-tailed unpaired t-tests were performed and the Holm-Šídák method was used to correct for multiple comparisons.

	Age	XX		XY		
		**Mean (%)**	**STD**	**Mean (%)**	**STD**	**Adj. P Value**
**LH+**	**P5**	10.01	2.97	9.8	0.91	0.935
	**P12**	43.51	9.12	45.88	1.29	0.935
	**P21**	56.31	0.5	51.94	6.8	0.790
	**7 weeks**	74.93	5.03	76.16	4.97	0.935
	**1 year**	76.16	2.49	69.16	3.63	0.232
**PRL+**	**P5**	2.13	1.76	4.00	2.43	0.791
	**P12**	2.80	0.44	2.70	1.23	0.896
	**P21**	7.50	5.58	2.49	0.37	0.791
	**7 weeks**	2.20	1.82	2.70	1.54	0.896
	**1 year**	5.17	0.35	6.20	1.20	0.791
**GH+**	**P5**	0.08	0.07	0.00	0.00	0.562
	**P12**	0.00	0.00	0.00	0.00	0.571
	**P21**	0.13	0.06	0.45	0.17	0.130
	**7 weeks**	0.17	0.06	0.65	0.69	0.571
	**1 year**	0.21	0.06	1.08	0.57	0.571
**TSH+**	**P5**	0.00	0.00	0.09	0.13	0.621
	**P12**	0.01	0.01	0.01	0.01	0.725
	**P21**	0.04	0.06	0.10	0.07	0.394
	**7 weeks**	0.16	0.01	0.31	0.30	0.988
	**1 year**	0.28	0.11	0.30	0.27	0.988
**POMC+**	**P5**	2.20	1.85	1.40	0.82	0.925
	**P12**	0.00	0.00	0.03	0.06	0.889
	**P21**	3.63	0.50	3.41	0.65	0.925
	**7 weeks**	6.27	4.08	7.00	1.73	0.925
	**1 year**	7.53	1.07	11.93	2.53	0.263

Supplementary Table 6: Cell-type signature used for single-cell RNAseq cluster identification.

CELL.SIGNATURE	GENE
Lactotroph	Prl
Lactotroph	Myoc
Lactotroph	Drd2
Lactotroph	Pou1f1
Somatotrophs	Gh
Somatotrophs	Ghrhr
Somatotrophs	Pou1f1
Thryotrophs	Tshb
Thryotrophs	Trhr
Thryotrophs	Dio2
Thryotrophs	Cga
Melanotrophs	Pomc
Melanotrophs	Pax7
Melanotrophs	Tbx19
Melanotrophs	Pcsk2
Corticotrophs	Crhr1
Corticotrophs	Tbx19
Corticotrophs	Pomc
Corticotrophs	Avpr1b
Gonadotrophs	Lhb
Gonadotrophs	Fshb
Gonadotrophs	Gnrhr
Gonadotrophs	Cga
Gonadotrophs	Nr5a1
Gonadotrophs	Foxp2
Endothelium	Cdh5
Endothelium	Angpt2
Endothelium	Pecam
Pericytes	Pdgfrb
Pericytes	Cspg4
Pericytes	Acta2
Macrophages	Cd68
Macrophages	Cd14
Macrophages	Ccr5
Stem cells	Sox2
Stem cells	Sox9
Stem cells	Hes1
Stem cells	Hey1
Stem cells	Fgfr1
Stem cells	Notch1
